# Comment on “New Antioxidant Drugs for Neonatal Brain Injury”

**DOI:** 10.1155/2015/384372

**Published:** 2015-09-21

**Authors:** Lajos Lakatos, György Balla

**Affiliations:** ^1^Department of Pediatrics, Kenezy Teaching Hospital, Bartók B. Street 2-26, Debrecen 4031, Hungary; ^2^Hungarian Academy of Sciences, Hungary; ^3^Department of Pediatrics, Faculty of Medicine, University of Debrecen, Nagyerdei Körút 98, Debrecen 4012, Hungary

In their thought-provoking and well-documented review, Tataranno et al. [1] have summarized the “new body” of knowledge about antioxidant drugs for neonatal brain injury. The authors, however, did not mention that D-Penicillamine (DPA) therapy is being used in the neonatal period (treatment in various forms of hyperbilirubinemia [2] and the prevention of retinopathy of prematurity (ROP), which, despite its peripheral location, the retina or neural portion of the eye, is actually part of the central nervous system [3, 4]) ever since 1973. Our recently published case reports, together with other convincing cases which participated in the long-term (28–40 years) follow-up, suggested that DPA therapy of newborn infants may have significant neuroprotective effects in cases jeopardized by bilirubin-induced neurologic dysfunction (BIND) or ROP [5]. This unexpected effect may be related to DPA capability to alter the nitric oxide (NO) system [6–9] and its strong antioxidant effects [10–12]. NO synthesized in the central nervous system produces a myriad of effects. For example, it plays a role in the control of blood flow, learning and memory, neurotransmitter release, gene expression, immune responsiveness, and cell survival. It is also implicated in numerous pathologies such as Alzheimer's disease, Huntington's disease, cerebral ischemia, and disorders of the basal ganglia caused by metals (Wilson's disease), bilirubin (BIND), or other pathologic conditions (Parkinsonism). The use of chelation therapy for nonmetal overload indications continues to be investigated. Furthermore, the mechanism of DPA in the reduction of serum bilirubin is based on the fact that this drug inhibits the rate limited enzyme (heme oxigenase) in heme metabolism [13]. Because those enzymes that play an important role in antioxidant defense and drug metabolism (peroxidases, catalase, and cytochrome P-450) are heme proteins, it can be assumed that in preventing hyperbilirubinemia, ROP, and oxygen toxicity, the mechanism of action of DPA is identical: the protection of biomembranes against lipid peroxidation caused by free radical. Low molecular weight disulfides are the major products of DPA metabolism in humans. The oxidation of DPA in vivo may also important in the mode of action of the drug through simultaneous reduction of oxygen species. Finally, we can say that DPA fulfills the criteria of a hybrid drug in the neonatal period by its ability to modulate both oxidative stress and NO pathway and can be a neuroprotective agent in the pathophysiology of neurologic dysfunction [14].
